# Determining Distinct Suicide Attempts From Recurrent Electronic Health Record Codes: Classification Study

**DOI:** 10.2196/46364

**Published:** 2024-01-08

**Authors:** Kate H Bentley, Emily M Madsen, Eugene Song, Yu Zhou, Victor Castro, Hyunjoon Lee, Younga H Lee, Jordan W Smoller

**Affiliations:** 1 Center for Precision Psychiatry Department of Psychiatry Massachusetts General Hospital Boston, MA United States; 2 Department of Psychiatry Harvard Medical School Boston, MA United States; 3 Psychiatric and Neurodevelopmental Genetics Unit Center for Genomic Medicine Massachusetts General Hospital Boston, MA United States; 4 Mass General Brigham Research Information Science and Computing Somerville, MA United States

**Keywords:** suicide, suicide attempt, self-injury, electronic health record, EHR, prediction, predictive model, predict, model, suicidal, informatics, automated rule, psychiatry, machine learning

## Abstract

**Background:**

Prior suicide attempts are a relatively strong risk factor for future suicide attempts. There is growing interest in using longitudinal electronic health record (EHR) data to derive statistical risk prediction models for future suicide attempts and other suicidal behavior outcomes. However, model performance may be inflated by a largely unrecognized form of “data leakage” during model training: diagnostic codes for suicide attempt outcomes may refer to prior attempts that are also included in the model as predictors.

**Objective:**

We aimed to develop an automated rule for determining when documented suicide attempt diagnostic codes identify distinct suicide attempt events.

**Methods:**

From a large health care system’s EHR, we randomly sampled suicide attempt codes for 300 patients with at least one pair of suicide attempt codes documented at least one but no more than 90 days apart. Supervised chart reviewers assigned the clinical settings (ie, emergency department [ED] versus non-ED), methods of suicide attempt, and intercode interval (number of days). The probability (or positive predictive value) that the second suicide attempt code in a given pair of codes referred to a distinct suicide attempt event from its preceding suicide attempt code was calculated by clinical setting, method, and intercode interval.

**Results:**

Of 1015 code pairs reviewed, 835 (82.3%) were nonindependent (ie, the 2 codes referred to the same suicide attempt event). When the second code in a pair was documented in a clinical setting other than the ED, it represented a distinct suicide attempt 3.3% of the time. The more time elapsed between codes, the more likely the second code in a pair referred to a distinct suicide attempt event from its preceding code. Code pairs in which the second suicide attempt code was assigned in an ED at least 5 days after its preceding suicide attempt code had a positive predictive value of 0.90.

**Conclusions:**

EHR-based suicide risk prediction models that include *International Classification of Diseases* codes for prior suicide attempts as a predictor may be highly susceptible to bias due to data leakage in model training. We derived a simple rule to distinguish codes that reflect new, independent suicide attempts: suicide attempt codes documented in an ED setting at least 5 days after a preceding suicide attempt code can be confidently treated as new events in EHR-based suicide risk prediction models. This rule has the potential to minimize upward bias in model performance when prior suicide attempts are included as predictors in EHR-based suicide risk prediction models.

## Introduction

Suicide is the tenth leading cause of death in the United States, with more than 48,000 suicide deaths annually [1]. Over the past 20 years, the suicide rate has increased by over 35% [2]. Most people who die by suicide have recently interacted with the health care system, with over half having a health care visit in the month prior to death [3,4]. Health care systems thus offer a key opportunity to identify people at high risk for suicide. Unfortunately, clinicians are poor at predicting who will make a suicide attempt [5] and traditionally studied risk factors perform no better than chance at predicting future suicidal behavior [6].

Recent work has focused on developing and validating machine learning models that use routinely collected electronic health record (EHR) data to predict future suicidal behavior [7]. Such models have demonstrated high levels of accuracy, exceeding that seen with clinician prediction and usual clinical risk factors [6,8-10]. EHR-based suicide risk prediction models, however, face one significant challenge that to date has not been adequately addressed. Suicide attempt is generally the outcome of interest in these models and is typically defined by *International Classification of Diseases* (ICD) diagnostic codes [11,12]. Within a given patient’s EHR, a suicide attempt code may be given multiple times across distinct health care encounters, often over very short periods of time (eg, days and weeks). Such “recurrent” codes may represent either distinct, new events (ie, multiple suicide attempts) or refer to the same event (ie, a single suicide attempt). The latter may occur when, for example, after making a suicide attempt, a patient has an emergency department (ED) visit followed by an inpatient hospitalization or outpatient follow-up encounters, with one (or multiple) suicide attempt codes assigned at each. In the absence of manual reviews of the narrative notes within patients’ EHRs, which cannot be performed at scale, it can be challenging to determine whether such recurrent suicide attempt codes, especially when documented over short time periods, refer to independent, distinct suicide attempts. Failure to make this important distinction can result in a form of “data leakage” in which the outcome to be predicted is included among features used for the prediction. This can result in substantial inflation of model performance [13].

To address this issue, some researchers have restricted model development to predict only the first occurrence of a suicide attempt code in a patient’s EHR [14-16]. This approach has a major limitation, however, in that a past suicide attempt is among the strongest known predictors of future suicidal behavior [17]. Thus, models that predict only the first documented suicide attempt ignore the subset of patients who may be at highest risk and thus of greatest clinical concern: those with a prior suicide attempt. Another approach is to include any previous suicide attempt codes as predictors of a subsequent suicide attempt code [18-21] thus including potential “repeat attempters” in these models. This approach, however, poses a significant risk of artificially inflating model performance if subsequent codes do not in fact refer to new suicide attempts. In other words, if a suicide attempt code instance used as an outcome actually indexes an attempt that was included a predictor, model performance will be inflated.

To minimize the risk of data leakage while retaining the option of including prior attempts as predictors, we aimed to develop an automated rule for determining whether recurrent suicide attempt codes in the EHR refer to distinct events. Such a rule might be based on relevant variables including clinical setting (eg, a suicide attempt code documented in the ED may be more likely to refer to a new suicide attempt event than one given in a non-ED setting), method (eg, suicide attempt codes that specify different methods may be more likely to refer to distinct events than codes specifying the same method), and time (eg, the more time elapsed between 2 suicide attempt codes, the less likely it may be that the codes refer to the same event). Here, we conducted a comprehensive manual EHR chart review to derive an automated rule that could identify criteria for selecting distinct suicide attempts with high confidence.

## Methods

### Data Source

The data source for this study was the Mass General Brigham (MGB) Research Patient Data Registry [22]. This registry covers 6.7 million patients treated in MGB-affiliated hospitals including the Massachusetts General Hospital and Brigham and Women’s Hospital in Boston.

### Ethics Approval

This research was approved by the MGB institutional review board, which granted a waiver of informed consent (protocol #2018P0001508).

### Case Definition and Inclusion Criteria

Details of the development of our EHR-based case definitions for suicide attempt in the MGB health care system are reported elsewhere [14,15]. In brief, we first identified candidate ICD*, Ninth Revision* (ICD-9) and *ICD, Tenth Revision* (ICD-10) codes that are likely to capture suicide attempts. Next, expert clinicians conducted manual chart reviews of 670 patients (over 3000 narrative notes) to determine a final set of codes that capture suicide attempts with a positive predictive value (PPV) of >0.70: for ICD-9, E95*, 965*, 967*, 969*, and 881*, and for ICD-10, X71*-X83*, T14.91*, T36*-T50* where the sixth character is 2 (except for T36.9, T37.9, T39.9, T41.4, T42.7, T43.9, T45.9, T47.9, and T49.9, where the fifth is 2) and T51*-T65* where the sixth character is 2 (except for T51.9, T52.9, T53.9, T54.9, T56.9, T57.9, T58.0, T58.1, T58.9, T59.9, T60.9, T61.0, T61.1, T61.9, T62.9, T63.9, T64.0, T64.8, and T65.9, where the fifth is 2).

For this study, we randomly selected a sample of 300 patients with 2 suicide attempt codes documented at least one but no more than 90 days apart (the “narrow sample”). This interval was chosen to capture codes that were given within a narrow time frame and thus potentially enriched for being “leaked” codes. In a sensitivity analysis, we randomly selected a second, smaller sample of 100 patients with 2 suicide attempt codes documented at least 1 day apart but with no other restrictions on intercode interval (the “broad sample”). A total of 31 patients appeared in both narrow and broad samples. Patients for whom we were unable to confidently locate the narrative notes corresponding to documented suicide attempt codes (eg, no narrative notes available within 30 days of the suicide attempt code date, narrative notes recorded on paper and never migrated to the EHR) were excluded after the sampling process.

### Procedure

Under the supervision of JWS (a senior clinician with expertise in the treatment of suicidal behavior), 2 study team members (EMM and ES) manually reviewed the EHR clinical encounter data (including narrative notes) relevant to each pair of suicide attempt codes (“code pair”) per sampled patient (1015 in the narrow sample and 300 in the broad sample; 1253 unique codes across the 2 samples). Each code pair comprised a given suicide attempt code and the immediately (temporally) preceding code in a patient’s EHR. All applicable code pairs per patient were examined (including other code pairs with >90-day intervals for patients in the narrow sample). Chart reviewers assigned the following variables to each code pair: (1) whether the code pair referred to 2 distinct suicide attempts (dichotomous variable indicating distinct or not distinct suicide attempts), (2) clinical setting in which each code in the pair was documented (dichotomous variable indicating ED or non-ED [eg, outpatient and inpatient] setting), (3) suicide attempt method of each code in the pair (categorical variable with 6 categories derived from previous literature: poisoning, cutting or piercing, hanging or strangulation or suffocation, jumping, firearm, and other [which included codes with no specified method]), and (4) time elapsed (in days) between codes in each pair [23]. When there were multiple encounters with suicide attempt codes on the same day, these variables were assigned to codes at the day level; see Table S1 in Multimedia Appendix 1 for an example of how we combined multiple same-day encounters.

### Data Analysis

We defined PPV as the probability that the second code in a pair of codes identified a new suicide attempt independent of the first code in the pair. To mimic the approach that would likely be taken in building predictive models, each code pair was treated independently (ie, we did not account for the nested nature of code pairs within patients). First, for the narrow sample, we calculated (in Excel [Microsoft]) PPVs and 95% CIs by clinical setting, suicide attempt method, and intercode interval, respectively. For clinical setting, we calculated the PPVs for 4 possible code pair types: (1) both codes documented in the ED (ED/ED), (2) first code ED and second code non-ED (ED/non-ED), (3) first code non-ED and second code ED (non-ED/ED), and (4) neither code ED (non-ED/non-ED). For suicide attempt method, we calculated the PPVs of 2 possible code pair types: (1) same suicide attempt method for codes in a pair and (2) different suicide attempt methods for codes in a pair. For intercode interval, we first calculated PPVs for all 7-day intervals from 1 to 91 days, followed by collapsing across intervals from 92 days on. We then calculated the PPVs for time intervals within each of the 6 (4 clinical settings and 2 suicide attempt methods) code pair types. To derive our proposed rule, we set our benchmark PPV to 0.90. For each of the 6 code pair types, we determined the minimum time elapsed between codes (ie, interval floor) at which the PPV was at least 0.90. For a sensitivity analysis, we computed the same series of PPVs for the broad sample.

## Results

### Descriptive Statistics

The mean number of suicide attempt codes per patient in the narrow sample was 3.38 (SD 4.62; range 1-47). A total of 225 (75%) patients had <4 codes and 281 (93.7%) had <10 codes. A total of 210 (20.7%) code pairs had a second code reflecting a subsequent encounter for a condition for which the patient had received active treatment (indicated by a seventh “D” character).

Regarding how often the codes in a pair referred to distinct suicide attempts, of the 300 patients in the narrow sample, only 81 (27%) had more than one confirmed (by manual chart review) suicide attempt captured by the reviewed code pairs. Of the 1015 code pairs, only 180 (17.7%) referred to 2 distinct suicide attempt events. Table S1 in Multimedia Appendix 1 presents an example of sampled codes (and the variables assigned to each code and code pair) for a deidentified patient.

For clinical setting, the most common code pair types were non-ED/non-ED (n=542, 53.4%) followed by ED/ED (n=274, 27%). Regarding the 749 total non-ED codes, the most commonly represented clinical setting was inpatient (n=411, 54.9% of all non-ED codes), followed by other or unclear setting (n=149, 19.9%codes), intensive or critical care units (n=134, 17.9% codes), and outpatient (n=55, 7.3% codes). For suicide attempt method, the majority of code pairs (n=766, 75.5%) comprised 2 codes that referred to the same method. The median interval between codes in each code pair, across all codes, was 1 day. Among code pairs that referred to distinct suicide attempt events, the median interval was 35 days.

### PPVs

#### Clinical Setting

Non-ED/ED code pairs (23 total code pairs) had the highest PPVs (0.96, 95% CI 0.87-1.04) for distinct suicide attempt events (Table 1). ED/ED pairs (274 total code pairs) had the second-highest PPVs (0.49, 95% CI 0.43-0.55). When the second code in a pair was assigned in a non-ED setting, PPVs were low (below 0.10).

In a sensitivity analysis, we excluded codes or encounters documented in inpatient settings with a prior code on the previous day from an inpatient or critical or intensive care setting. For example, if a patient was given suicide attempt codes on three consecutive days in an inpatient setting, we only used the day 1 code. This resulted in 792 (versus 1015) analyzed code pairs. The results were overall very similar to when we did not exclude contiguous inpatient codes (Multimedia Appendix 2).

**Table 1:** Code pairs in the narrow sample defined by the clinical setting (ED^a^ or non-ED) of the first and second codes in each pair.

First code clinical setting	Second code clinical setting	Code pairs (percentage of all code pairs), n (%)	Interval between codes (days), median (Q1^b^, Q3^c^)		Interval between codes (days), mean (SD)		Code pairs referring to distinct attempts, n		PPV^d^ (95% CI)	
Non-ED	Non-ED	542 (53.4)	1 (1, 1)		3.58 (8.64)		14		0.03 (0.01-0.04)	
ED	Non-ED	176 (17.3)	1 (1, 3)		6.47 (34.23)		10		0.06 (0.02-0.09)	
Non-ED	ED	23 (2.3)	52 (13, 125)		154.09 (286.63)		22		0.96 (0.87-1.04)	
ED	ED	274 (27)	5 (1, 36)		53.79 (211.77)		134		0.49 (0.43-0.55)	
Overall		1015		1		21.05 (122.43)		180		0.18 (0.15-0.20)

^a^ED: emergency department.

^b^Q1: first quartile.

^c^Q3: third quartile.

^d^PPV: positive predictive value.

#### Suicide Attempt Method

For suicide attempt method (same versus different method for 2 codes in a code pair), the PPVs were below 0.25 (Table 2). Table S6 in Multimedia Appendix 3 shows PPVs for each combination of the 6 aforementioned specific method categories derived from previous literature. All PPVs for strata containing more than 1 code pair were at or below 0.50.

**Table 2:** Code pairs defined by whether the first and second codes referred to the same or a different suicide attempt method.

First and second code	Code pairs (percentage of all code pairs), n (%)	Code pairs referring to distinct attempts, n	PPV^a^ (95% CI)
Same method	766 (75.5)	128	0.17 (0.14-0.19)
Different method	249 (24.5)	52	0.21 (0.16-0.26)
Overall	1015	180	0.18 (0.15-0.20)

^a^PPV: positive predictive value.

#### Intercode Interval

Table 3 presents PPVs for code pairs broken down by 7-day (week-long) intervals; the majority (n=797, 78.5%) of code pairs had an intercode interval of 7 days or less. The more days elapsed between 2 codes, the larger the PPV (and, fewer code pairs per strata). Table S7 in Multimedia Appendix 4 presents PPVs for code pairs broken down by interval and clinical setting (non-ED/non-ED, ED/non-ED, non-ED/ED, ED/ED), and Table S8 in Multimedia Appendix 5 presents PPVs for code pairs broken down by interval and suicide attempt method (same versus different). In another sensitivity analysis, given that ICD-9 is no longer used, we also computed all PPVs reported in Tables 1-3 when excluding code pairs with at least one ICD-9 coded event. The same pattern of findings held, with 95% CIs for all PPVs overlapping with those in Tables 1-3.

**Table 3:** Code pairs defined by intercode interval.

Intercode interval	Code pairs (percentage of all code pairs), n (%)	Code pairs referring to distinct attempts, n	PPV^a^ (95% CI)
1-7 days	797 (78.5)	31	0.04 (0.03-0.05)
8-14 days	48 (4.7)	19	0.40 (0.26-0.53)
15-21 days	31 (3)	17	0.55 (0.37-0.72)
22-28 days	20 (2)	14	0.70 (0.50-0.90)
29-35 days	17 (1.7)	10	0.59 (0.35-0.82)
36-42 days	17 (1.7)	15	0.88 (0.73-1.04)
43-49 days	10 (1)	8	0.80 (0.55-1.05)
50-56 days	18 (1.8)	14	0.78 (0.59-0.97)
57-63 days	5 (4.9)	3	0.60 (0.17-1.03)
64-70 days	7 (0.7)	6	0.86 (0.60-1.12)
71-77 days	2 (0.2)	2	1.00 (1.00-1.00)
78-84 days	9 (0.9)	9	1.00 (1.00-1.00)
85-91 days	6 (0.6)	6	1.00 (1.00-1.00)
92+ days	28 (2.7)	26	0.93 (0.83-1.02)
Overall	1015	180	0.18 (0.15-0.20)

^a^PPV: positive predictive value.

**Figure 1:**
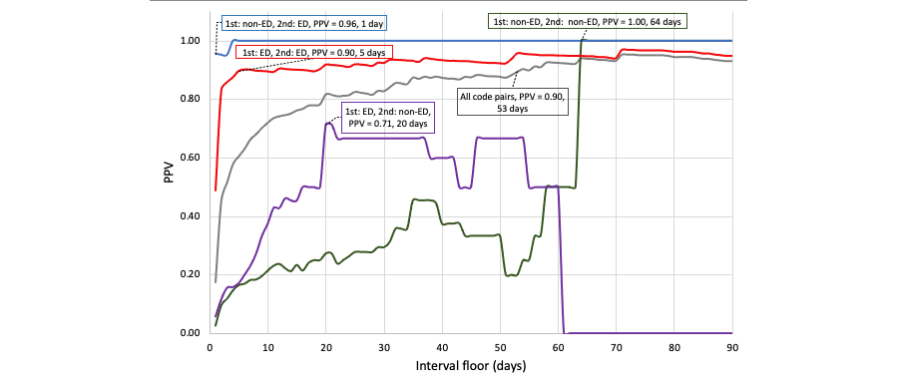
PPVs for interval floors by code pair types defined by clinical setting. The labeled data points indicate the interval floor at which the PPV was at least 0.90 (or the maximum PPV). Gray lines reflect PPVs for interval floors across all code pair types. Red lines refer to code pairs documented in ED (first code) and ED (second code) settings (ED/ED). Blue lines are non-ED/ED code pairs; purple lines ED/non-ED; and green lines non-ED/non-ED. ED: emergency department; PPV: positive predictive value.

**Figure 2:**
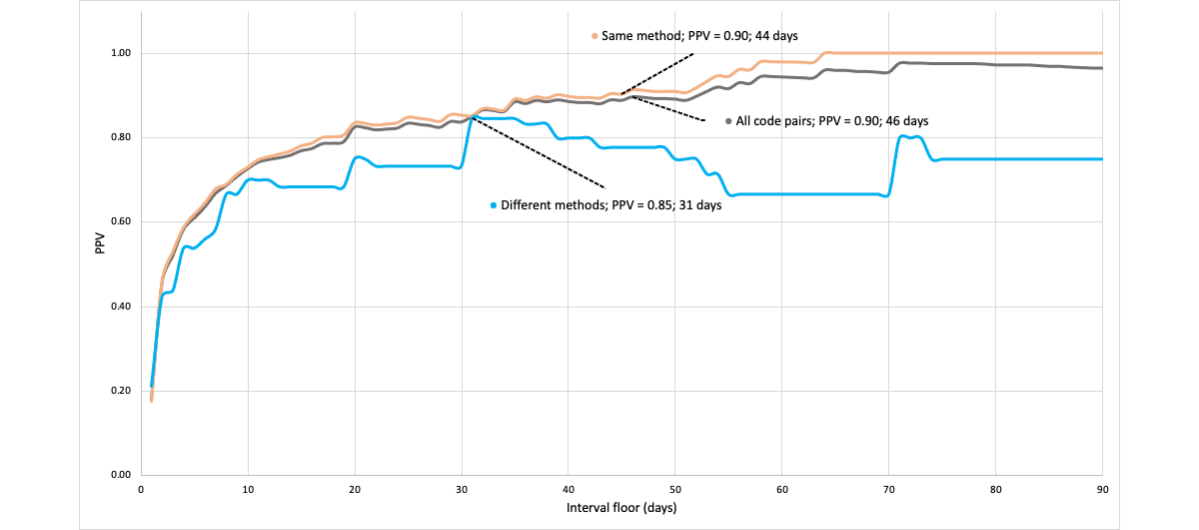
PPVs for interval floors by code pair types defined by suicide attempt method with intervals greater than or equal to the plotted interval floor values. The labeled data points indicate the interval floor at which the PPV was at least 0.90 (or the maximum PPV). Gray lines reflect PPVs for interval floors across all code pair types. Orange lines refer to code pairs in which the 2 codes refer to the same suicide attempt method. Blue lines refer to code pairs in codes in the pairs referring to different suicide attempt methods. PPV: positive predictive value.

As shown in Figure 1, across all code pairs, pairs with an interval of at least 53 days had a PPV of 0.90 (range 0.88-0.93). The interval floors meeting our benchmark PPV (at least 0.90) within each of the 6 code pair types are also labeled in Figure 1 (clinical setting) and Figure 2 (suicide attempt method). For non-ED/ED code pairs (23 code pairs), an interval floor of 1 day had a PPV of 0.96. When both codes were assigned in the ED (271 code pairs), PPV reached 0.90 when the intercode interval was at least 5 days. When the second code in a pair was documented in an ED (regardless of the setting in which the first code was documented), PPV was 0.91 when the intercode interval was 5 days (the PPV was 0.89 for 4 days). Thus, whenever the second code in a pair was documented in an ED at least 5 days after the previous code, the probability that the second code referred to an independent suicide attempt was at least 90%.

### Sensitivity Analysis: Broad Sample

Results from the same series of analyses in the broad sample are presented in Multimedia Appendix 6. Of the 100 patients, 45 (45%) had more than 1 confirmed suicide attempt. Of the 300 code pairs, 86 (28.7%) referred to 2 distinct suicide attempts. The median interval between codes in each pair was also 1 day. Among code pairs that referred to distinct suicide attempts, the median interval was 133 days. Overall, we found a similar pattern of PPVs (in almost all cases overlapping 95% CIs) to those from the narrow sample. Across all code pairs in the broad sample, those with an interval of at least 37 days had a PPV of 0.90 (range 0.87-0.93). When both codes were given in the ED (86 pairs), PPV reached 0.90 when the interval was at least 2 days.

## Discussion

### Primary Findings

Machine learning suicide risk prediction models that leverage routinely collected EHR data can outperform clinician assessment [8] and have the potential to improve how patients at risk for suicide are identified and treated. These models are typically trained using ICD codes to label suicide attempts. An under-appreciated challenge when building these models, however, is that ICD codes indexing a single suicide attempt are often used repeatedly across multiple encounters. This could create a substantial problem for models that incorporate prior suicide attempts, an established risk factor, in predicting subsequent attempts or suicidal behavior.

Some investigators side-step this issue by restricting model predictions to only the first occurrence of a suicide attempt code. This approach, however, limits the utility of prediction models by ignoring prior attempts, the best-known risk factor for suicidal behavior, and limiting their application to a subset of those at risk; prior studies indicate that nearly one-quarter of those who engage in deliberate self-harm have recurrent episodes within 3 years [24]. Here we aimed to develop a portable, automated rule for determining when recurrent suicide attempt codes refer to distinct suicide attempt events in a patient’s history. Based on chart review of clinical encounters corresponding to 1015 unique ICD code pairs, we found that, for patients with more than 1 documented suicide attempt code, repeat codes most often (>80% of the time) reflected nonindependent events, underscoring the high frequency of “leaked” suicide attempt codes. When collapsing across all clinical settings, repeat codes needed to be documented at least 53 days after the preceding code in order to refer (with probability >90%) to a new, distinct suicide attempt. However, repeat codes documented *in an ED* *at least 5 days* after the preceding suicide attempt code were likely (probability >90%) to refer to a new, distinct suicide attempt.

The most informative variables for determining whether recurrent suicide attempt codes referred to distinct suicide attempts were the clinical setting in which the codes were documented and the time elapsed between codes. First, regarding clinical setting, when a suicide attempt code was documented in an ED after the preceding code, it referred to a new suicide attempt more than half the time. Suicide attempt codes documented in non-ED settings, accounting for most of the second codes among all code pairs, however, were highly unlikely to refer to a new suicide attempt (probability <5%). This may be due to the fact that the vast majority (nearly three-quarters) of non-ED codes occurred in inpatient or intensive or critical care units, where patients may be treated over the course of several days or longer, potentially accumulating multiple suicide attempt codes that all refer to the same index event that may have prompted inpatient or intensive treatment. This pattern of findings, for one, highlights the considerable risk of treating all recurrent suicide attempt codes (especially those from non-ED settings) as distinct events, and the potential importance of using a simple rule, such as that proposed here, to identify probable distinct suicide attempt events.

Along these lines, the more time elapsed between 2 suicide attempt codes, the more likely it was the codes referred to distinct events. Combining these 2 variables—clinical setting and time elapsed—provided a simple rule for determining whether recurrent suicide attempt codes refer to distinct events with at least 90% probability. Although the accuracy of our proposed rule (at least 5 days elapsed between a code given in the ED and the preceding code) may differ in other health care systems, we recommend that others consider taking into account these 2 variables when incorporating recurrent suicide attempt codes in EHR-based suicide risk prediction models.

Perhaps surprisingly, whether the coded suicide attempt method for 2 codes in a pair was the same or different did not provide value in identifying distinct suicide attempt events. However, in the relatively small proportion of code pairs (24.5%) that referred to different methods, the most common “profile” was 1 code with a specific method (eg, poisoning and cutting or piercing) and the other code with method categorized as “other” (*not* a different specific method); notably, the “other” category included codes lacking any specified method. Thus, the fact that method did not help identify distinct events may largely reflect inconsistencies in how or whether the suicide attempt method is coded by providers. In contrast, neither of the other 2 variables examined (clinical setting nor intercode interval) should be impacted by irregular coding practices, and thus may also be more scalable and reliable for other health care systems planning to use this or a similar rule.

Our derived rule (at least 5 days elapsed between a code from the ED and the preceding code) may have more impact on certain suicide-related prediction tasks than others. For example, it may be especially relevant when estimating patients’ risk of repeat suicidal behavior, for example after an ED visit for suicidal behavior, which could influence clinical decision-making at the point of care (eg, about discharge home or to outpatient care versus hospitalization). This rule may have less impact for other related prediction tasks, such as estimating patients’ risk of suicidal behavior after nonsuicide-related outpatient visits or broader population-based prediction efforts [25]. These results may also be less relevant for models that solely predict fatal self-harm or suicide deaths [26,27]. Future work should systematically evaluate the performance and clinical utility of models that do and do not incorporate the proposed rule for incorporating recurrent suicide attempt codes across a range of prediction goals and clinical contexts.

Our results must be considered in the context of a few key limitations. First, some of the sampled patients may have presented to hospitals outside of the MGB system for suicide attempts. In these cases, the corresponding diagnostic codes and contextual information were either unavailable or only sporadically recorded in narrative notes at subsequent clinical encounters within MGB. We also excluded sampled patients for whom chart reviewers could not confidently match data pulled from the MGB Research Patient Data Registry to the narrative notes.

### Conclusions

This analysis indicates that EHR-based suicide attempt prediction models that include ICD codes for prior attempts as a predictor may be highly susceptible to bias due to data leakage in model training. Our proposed rule for circumventing this issue should minimize this bias and its inflationary effect on model performance metrics. The key variables included in our rule (clinical setting and time elapsed between codes) are widely available in health system data warehouses and should be easily integrated into EHR-based models. It is also possible that the approach taken in this study may be relevant for developing and refining machine learning models aimed to predict other episodic events of interest that can be repeatedly documented in the health record, such as unintentional overdose, domestic abuse, or episodes of violence. If effectively implemented into existing and future suicide risk prediction models, this rule could increase the robustness and validity of machine-learning based approaches to identifying the individuals at highest risk for suicide, and ultimately advance suicide prevention efforts in health care contexts on a large scale.

## Appendix

Multimedia Appendix 1 Sampled codes or code pairs and designations per manual chart review for an example (deidentified) patient.

Multimedia Appendix 2 Sensitivity analysis (excluding contiguous codes from inpatient settings).

Multimedia Appendix 3 Code pairs in the narrow sample defined by specific category of suicide attempt method (poisoning, cutting or piercing, hanging or strangulation or suffocation, jumping, firearm, or other) of the first and second code in each code pair.

Multimedia Appendix 4 Code pairs in the narrow sample defined by both the clinical setting (ED or non-ED) of and the interval (in days) between the first and second codes in each pair.

Multimedia Appendix 5 Code pairs in the narrow sample defined by both suicide attempt method (same or different) and the interval (in days) between first and second codes in each pair.

Multimedia Appendix 6 Sensitivity analysis (results for broad sample).

## Abbreviations

ED: emergency department
EHR: electronic health record
ICD: International Classification of Diseases
MGB: Mass General Brigham
PPV: positive predictive value

## Data Availability

The data used in this study cannot be made publicly available due to restrictions relating to the use of EHR data.
