# Supplementary material for: Determining Distinct Suicide Attempts From Recurrent Electronic Health Record Codes: Classification Study
Source: JMIR Form Res. 2024 Jan 8;8:e46364. doi: 10.2196/46364 (PMC10804255; doi:10.2196/46364)
Supplement: Multimedia Appendix 1 [file formative_v8i1e46364_app1.docx]

| **Day** | **Description** | **ICD-10 Code*** | **Suicide Method** | **Setting** | **Code Pair Setting**  **(First Code/ Second Code)*** | **Second Code in Pair Refers to Distinct Event from First Code?*** | **Suicide Attempt Events** |
| --- | --- | --- | --- | --- | --- | --- | --- |
| Day 1 | Patient arrives in the ED with minor arm lacerations and states they are having suicidal thoughts | X78.9 | Cutting or Piercing | ED | N/A (First sampled code) | N/A (First sampled code) | Event #1 |
| Day 2 | Patient remains in ED while clinician looks for a psych bed; is admitted to inpatient unit late on Day 2 | X78.9 | Cutting or Piercing | ED | ED/ED | No |  |
| Day 3 | Patient remains on inpatient unit | X78.9 | Cutting or Piercing | Inpatient Psychiatry | ED/Non-ED | No |  |
| Day 8 | Patient is discharged from inpatient psychiatry | X78.9 | Cutting or Piercing | Inpatient Psychiatry | Non-ED/Non-ED | No |  |
| Day 320 | Patient arrives in the ED with superficial scratches on their arm. Patient is discharged the same day | X78.9 | Cutting or Piercing | ED | Non-ED/ED | **Yes** | Event #2 |
| Day 327 | Patient returns to ED and states they are having worsening suicidal thoughts. Patient is intoxicated and has superficial scratches on their arm. | T51.92, X78.9 | Poisoning, Cutting or Piercing | ED | ED/ED | **Yes** | Event #3 |
| Day 328 | Patient remains in ED while clinician looks for a psychiatry bed | T51.92 | Poisoning | ED | ED/ED | No |  |
| Day 329 | Patient is admitted to inpatient psychiatry | T51.92 | Poisoning | Inpatient Psychiatry | ED/Non-ED | No |  |
| Day 361 | Patient arrives in ED; they have seriously injured themselves with a knife and state they want to die | X78.1 | Cutting or piercing | ED | Non-ED/ED | **Yes** | Event #4 |
| Day 362 | Patient is admitted to inpatient medicine | X78.1 | Cutting or piercing | Inpatient Medicine | ED/Non-ED | No |  |
| Day 367 | Patient is discharged from inpatient medicine to inpatient psychiatry | X78.1 | Cutting or piercing | Inpatient Psychiatry | Non-ED/Non-ED | No |  |
| Day 368 | Patient remains in inpatient psychiatry | X78.1 | Cutting or piercing | Inpatient Psychiatry | Non-ED/Non-ED | No |  |
| Day 371 | Patient is discharged from inpatient psychiatry | X78.1 | Cutting or piercing | Inpatient Psychiatry | Non-ED/Non-ED | No |  |
| *Note.* Details have been changed for purposes of patient de-identification. *For each row, the corresponding code pair consists of the preceding row’s code (first code in the pair) and that row’s code (second code in the pair). | | | | | | | |

**Table S1.** Sampled codes/code pairs and designations per manual chart review for an example (de-identified) patient.
