# Supplementary material for: Determining Distinct Suicide Attempts From Recurrent Electronic Health Record Codes: Classification Study
Source: JMIR Form Res. 2024 Jan 8;8:e46364. doi: 10.2196/46364 (PMC10804255; doi:10.2196/46364)
Supplement: Multimedia Appendix 2 [file formative_v8i1e46364_app2.docx]

**Supplemental File: Sensitivity Analysis**

**(Excluding Contiguous Codes from Inpatient Settings)**

In this sensitivity analysis, we excluded codes/encounters documented in inpatient settings with a prior code on the previous day from an inpatient or critical/intensive care setting. For example, if a patient had a suicide attempt code from an inpatient or critical care setting on day 1, followed by another suicide attempt code from an inpatient setting on day 2, we excluded the code from day 2 in this sensitivity analysis. If the patient then had another suicide attempt code on day 10 in an inpatient setting, however, we included the code from day 10. This resulted in removing 223 code pairs from the Narrow Sample (for a total of 792 analyzed code pairs versus 1015 from the main sets of analyses). Of these 792 code pairs, 22.6% referred to distinct events.

**Table S2.** Code pairs in the Narrow Sample defined by the clinical settings of the first and second codes in each pair (excluding contiguous codes from inpatient settings).

| **First code clinical setting** | **Second code clinical setting** | **Number of code pairs (% of all code pairs)** | **Median interval between codes (days)** | **Mean interval between codes (days)** | **Code pairs referring to distinct attempts** | **PPV**  **(95% CI)** |
| --- | --- | --- | --- | --- | --- | --- |
| **Non-ED** | **Non-ED** | 319 (40.3%) | 1  (Q1: 1; Q3: 4) | 5.36  (SD: 10.92) | 13 | 0.04  (0.02, 0.06) |
| **ED** | **Non-ED** | 176 (22.2%) | 1 (Q1: 1; Q3: 3) | 6.47  (SD: 34.23) | 10 | 0.06  (0.02, 0.09) |
| **Non-ED** | **ED** | 23 (2.9%) | 52 (Q1: 13; Q3: 125) | 154.09  (SD: 286.63) | 22 | 0.96  (0.87, 1.04) |
| **ED** | **ED** | 274 (34.6%) | 5 (Q1: 1; Q3: 36) | 53.79  (SD: 211.77) | 134 | 0.49  (0.43, 0.55) |
| **Overall** | | 792 | 1 (Q1: 1; Q3: 10) | 26.68  (SD: 138.10) | 179 | 0.23  (0.20, 0.26) |

**Table S3.** Code pairs in the Narrow Sample defined by whether the first and second codes referred to the same or a different suicide attempt method (excluding contiguous codes from inpatient settings).

| **First and second code** | **Number of code pairs**  **(% of all code pairs)** | **Code pairs referring to distinct attempts** | **PPV**  **(95% CI)** |
| --- | --- | --- | --- |
| **Same method** | 594 (75.0%) | 127 | 0.21  (0.18, 0.25) |
| **Different method** | 198 (25.0%) | 52 | 0.26  (0.20, 0.32) |
| **Overall** | 792 | 179 | 0.23  (0.20, 0.26) |

**Table S4.** Code pairs in the Narrow Sample defined by inter-code interval (excluding contiguous codes from inpatient settings).

| **Inter-code interval** | **Number of code pairs (% of all code pairs)** | **Code pairs referring to distinct attempts** | **PPV**  **(95% CI)** |
| --- | --- | --- | --- |
| **1-7 days** | 574 (72.5%) | 30 | 0.05  (0.30, 0.70) |
| **8-14 days** | 48 | 19 | 0.40  (0.26, 0.54) |
| **15-21 days** | 31 | 17 | 0.55  (0.37, 0.73) |
| **22-28 days** | 20 | 14 | .70  (0.50, 0.90) |
| **29-35 days** | 17 | 10 | .59  (0.35, 0.83) |
| **36-42 days** | 17 | 15 | .88  (0.72, 1.04) |
| **43-49 days** | 10 | 8 | .80  (0.55, 1.05) |
| **50-56 days** | 18 | 14 | .78  (0.58, 0.98) |
| **57-63 days** | 5 | 3 | .60  (0.17, 1.03) |
| **64-70 days** | 7 | 6 | .86  (0.61, 1.11) |
| **71-77 days** | 2 | 2 | 1.00  (1.00, 1.00) |
| **78-84 days** | 9 | 9 | 1.00  (1.00, 1.00) |
| **85-91 days** | 6 | 6 | 1.00  (1.00, 1.00) |
| **92+ days** | 28 | 28 | 0.93  (0.83, 1.03) |
| **Total** | 792 | 179 | 0.23  (0.20, 0.26) |

**Table S5.** Interval floor (in days) at which PPV was at least .90 (or the maximum PPV) by setting (excluding contiguous codes from inpatient settings)

| **Code Pair Type** | **Interval floor at which**  **PPV > .90 (or maximum PPV)** |
| --- | --- |
| **Non-ED/Non-ED** | 64 days (PPV = 1.00) |
| **ED/Non-ED** | 20 days (PPV = 0.71) |
| **Non-ED/ED** | 1 day (PPV = 0.96) |
| **ED/ED** | 5 days (PPV = 0.90) |
| **All Code Pairs** | 53 days (PPV = 0.90) |
