# Supplementary material for: Determining Distinct Suicide Attempts From Recurrent Electronic Health Record Codes: Classification Study
Source: JMIR Form Res. 2024 Jan 8;8:e46364. doi: 10.2196/46364 (PMC10804255; doi:10.2196/46364)
Supplement: Multimedia Appendix 3 [file formative_v8i1e46364_app3.docx]

**Table S6.** Code pairs in the Narrow Sample defined by specific category of suicide attempt method (poisoning, cutting/piercing, hanging/strangulation/suffocation, jumping, firearm, or other) of the first and second code in each code pair.

|  | | **1st code: Poisoning** | **1st code: Cutting/**  **piercing** | **1st code: Hanging/**  **strangulation/**  **suffocation** | **1st code: Jumping** | **1st code: Firearm** | **1st code: Other** |
| --- | --- | --- | --- | --- | --- | --- | --- |
| **2nd code: Poisoning** | **Count** | 489 | 42 | 2 | 0 | 1 | 72 |
|  | **PPV**  **(95% CI)** | 0.09  (0.07 - 0.12) | 0.19  (0.07 - 0.31) | 0.00  (0.00 - 0.00) | N/A | 1.00  (1.00 - 1.00) | 0.11  (0.04 - 0.18) |
| **2nd code: Cutting/**  **Piercing** | **Count** | 43 | 208 | 1 | 1 | 0 | 30 |
|  | **PPV**  **(95% CI)** | 0.16  (0.05 - 0.27) | 0.16  (0.11 - 0.21) | 0.00  (0.00 - 0.00) | 1.00  (1.00 - 1.00) | N/A | 0.13  (0.01 - 0.25) |
| **2nd code: Hanging/**  **strangulation/ suffocation** | **Count** | 1 | 2 | 6 | 1 | 0 | 5 |
|  | **PPV**  **(95% CI)** | 0.00  (0.00 - 0.00) | 0.00  (0.00 - 0.00) | 0.00  (0.00 - 0.00) | 0.00  (0.00 - 0.00) | NA | 0.00  (0.00 - 0.00) |
| **2nd code: Jumping** | **Count** | 0 | 6 | 0 | 5 | 0 | 3 |
|  | **PPV**  **(95% CI)** | NA | 0.50  (0.10 - 0.90) | NA | 0.40  (-0.03 - 0.83) | NA | 0.33  (-0.20 - 0.87) |
| **2nd code: Firearm** | **Count** | 1 | 0 | 0 | 0 | 5 | 4 |
|  | **PPV**  **(95% CI)** | 0.00  (0.00 - 0.00) | NA | NA | NA | 0.40  (-0.03 - 0.83) | 0.50  (0.01 - 0.99) |
| **2nd code:**  **Other** | **Count** | 54 | 31 | 2 | 4 | 6 | 168 |
|  | **PPV**  **(95% CI)** | 0.07  (0.00 - 0.14) | 0.13  (0.01 - 0.25) | 0.00  (0.00 - 0.00) | 0.00  (0.00 - 0.00) | 0.33  (-0.04 - 0.71) | 0.10  (0.05 - 0.14) |

*Note.* Count refers to the number of code pairs in each strata. The “other” category included codes with no specific suicide attempt method. There were a total of 66 patients who received at least 2 codes referring to different suicide attempt methods on the same day. For the primary sets of analyses for suicide attempt method (Table 2), we combined codes at the day level in order to assign code pairs to “same method” or “different method.” For example, if there were a poisoning code and a cutting code on day 1, followed by a poisoning code on day 2, we would analyze only one code pair and assigned it to “different method.” However, for this supplemental table above presenting more granularity in suicide attempt code methods, we would analyze two distinct code pairs: poisoning-poisoning and poisoning-cutting. This results in a slightly larger number of total code pairs here (1,193) than that included in Table 2 (1,015).
