# Supplementary material for: Determining Distinct Suicide Attempts From Recurrent Electronic Health Record Codes: Classification Study
Source: JMIR Form Res. 2024 Jan 8;8:e46364. doi: 10.2196/46364 (PMC10804255; doi:10.2196/46364)
Supplement: Multimedia Appendix 4 [file formative_v8i1e46364_app4.docx]

**Table S7.** Code pairs in the Narrow Sample defined by both the clinical setting (ED or Non-ED) of and the interval (in days) between the first and second codes in each pair.

| **First and second code** | | **Number of code pairs** | **Code pairs referring to distinct attempts** | **PPV** | **95% CI (lower limit)** | **95% CI (upper limit)** |
| --- | --- | --- | --- | --- | --- | --- |
| **Setting**  **(first code- second code)** | **Interval** |  | | | | |
| **Not ED-**  **Not ED** | 1-7 days | 493 | 5 | 0.01 | 0.00 | 0.02 |
|  | 8-14 days | 19 | 2 | 0.11 | -0.03 | 0.24 |
|  | 15-21 days | 9 | 2 | 0.22 | -0.05 | 0.49 |
|  | 22-28 days | 4 | 0 | 0.00 | 0.00 | 0.00 |
|  | 29-35 days | 6 | 0 | 0.00 | 0.00 | 0.00 |
|  | 36-42 days | 3 | 2 | 0.67 | 0.13 | 1.20 |
|  | 43-49 days | 2 | 1 | 0.50 | -0.19 | 1.19 |
|  | 50-56 days | 3 | 1 | 0.33 | -0.20 | 0.87 |
|  | 57-63 days | 2 | 0 | 0.00 | 0.00 | 0.00 |
|  | 64-70 days | 0 | 0 | NA | NA | NA |
|  | 71-77 days | 0 | 0 | NA | NA | NA |
|  | 78-84 days | 0 | 0 | NA | NA | NA |
|  | 85-91 days | 1 | 1 | 1.00 | 1.00 | 1.00 |
|  | 92+ days | 0 | 0 | NA | NA | NA |
| **ED-**  **Not ED** | 1-7 days | 154 | 4 | 0.03 | 0.00 | 0.05 |
|  | 8-14 days | 11 | 1 | 0.09 | -0.08 | 0.26 |
|  | 15-21 days | 5 | 1 | 0.20 | -0.15 | 0.55 |
|  | 22-28 days | 0 | 0 | NA | NA | NA |
|  | 29-35 days | 0 | 0 | NA | NA | NA |
|  | 36-42 days | 2 | 2 | 1.00 | 1.00 | 1.00 |
|  | 43-49 days | 1 | 0 | 0.00 | 0.00 | 0.00 |
|  | 50-56 days | 1 | 1 | 1.00 | 1.00 | 1.00 |
|  | 57-63 days | 1 | 1 | 1.00 | 1.00 | 1.00 |
|  | 64-70 days | 0 | 0 | NA | NA | NA |
|  | 71-77 days | 0 | 0 | NA | NA | NA |
|  | 78-84 days | 0 | 0 | NA | NA | NA |
|  | 85-91 days | 0 | 0 | NA | NA | NA |
|  | 92+ days | 1 | 0 | 0.00 | 0.00 | 0.00 |
| **Not ED-**  **ED** | 1-7 days | 5 | 4 | 0.80 | 0.45 | 1.15 |
|  | 8-14 days | 2 | 2 | 1.00 | 1.00 | 1.00 |
|  | 15-21 days | 1 | 1 | 1.00 | 1.00 | 1.00 |
|  | 22-28 days | 1 | 1 | 1.00 | 1.00 | 1.00 |
|  | 29-35 days | 0 | 0 | NA | NA | NA |
|  | 36-42 days | 0 | 0 | NA | NA | NA |
|  | 43-49 days | 1 | 1 | 1.00 | 1.00 | 1.00 |
|  | 50-56 days | 3 | 3 | 1.00 | 1.00 | 1.00 |
|  | 57-63 days | 0 | 0 | NA | NA | NA |
|  | 64-70 days | 1 | 1 | 1.00 | 1.00 | 1.00 |
|  | 71-77 days | 0 | 0 | NA | NA | NA |
|  | 78-84 days | 1 | 1 | 1.00 | 1.00 | 1.00 |
|  | 85-91 days | 0 | 0 | NA | NA | NA |
|  | 92+ days | 8 | 8 | 1.00 | 1.00 | 1.00 |
| **ED-ED** | 1-7 days | 145 | 18 | 0.12 | 0.07 | 0.18 |
|  | 8-14 days | 16 | 14 | 0.88 | 0.71 | 1.04 |
|  | 15-21 days | 16 | 13 | 0.81 | 0.62 | 1.00 |
|  | 22-28 days | 15 | 13 | 0.87 | 0.69 | 1.04 |
|  | 29-35 days | 11 | 10 | 0.91 | 0.74 | 1.08 |
|  | 36-42 days | 12 | 11 | 0.92 | 0.76 | 1.07 |
|  | 43-49 days | 6 | 6 | 1.00 | 1.00 | 1.00 |
|  | 50-56 days | 11 | 9 | 0.82 | 0.59 | 1.05 |
|  | 57-63 days | 2 | 2 | 1.00 | 1.00 | 1.00 |
|  | 64-70 days | 6 | 5 | 0.83 | 0.54 | 1.13 |
|  | 71-77 days | 2 | 2 | 1.00 | 1.00 | 1.00 |
|  | 78-84 days | 8 | 8 | 1.00 | 1.00 | 1.00 |
|  | 85-91 days | 5 | 5 | 1.00 | 1.00 | 1.00 |
|  | 92+ days | 19 | 18 | 0.95 | 0.85 | 1.05 |
| **Overall** |  | 1015 | 180 | 0.18 | 0.15 | 0.20 |
