# Supplementary material for: Determining Distinct Suicide Attempts From Recurrent Electronic Health Record Codes: Classification Study
Source: JMIR Form Res. 2024 Jan 8;8:e46364. doi: 10.2196/46364 (PMC10804255; doi:10.2196/46364)
Supplement: Multimedia Appendix 5 [file formative_v8i1e46364_app5.docx]

**Table S8.** Code pairs in the Narrow Sample defined by both suicide attempt method (same or different) and the interval (in days) between first and second codes in each pair.

| **First and second code** | | **Number of code pairs** | **Code pairs referring to distinct attempts** | **PPV** | **95% CI (lower limit)** | **95% CI (upper limit)** |
| --- | --- | --- | --- | --- | --- | --- |
| **Method** | **Interval** |  | | | | |
| **Same method** | 1-7 days | 610 | 25 | 0.04 | 0.02 | 0.06 |
|  | 8-14 days | 38 | 14 | 0.37 | 0.21 | 0.53 |
|  | 15-21 days | 20 | 10 | 0.50 | 0.28 | 0.72 |
|  | 22-28 days | 16 | 10 | 0.62 | 0.38 | 0.86 |
|  | 29-35 days | 11 | 6 | 0.55 | 0.26 | 0.84 |
|  | 36-42 days | 8 | 7 | 0.11 | 0.66 | 1.10 |
|  | 43-49 days | 8 | 6 | 0.75 | 0.46 | 1.04 |
|  | 50-56 days | 12 | 9 | 0.75 | 0.51 | 0.99 |
|  | 57-63 days | 5 | 3 | 0.60 | 0.17 | 1.03 |
|  | 64-70 days | 5 | 5 | 1.00 | 1.00 | 1.00 |
|  | 71-77 days | 1 | 1 | 1.00 | 1.00 | 1.00 |
|  | 78-84 days | 8 | 8 | 1.00 | 1.00 | 1.00 |
|  | 85-91 days | 4 | 4 | 1.00 | 1.00 | 1.00 |
|  | 92+ days | 20 | 20 | 1.00 | 1.00 | 1.00 |
| **Different method** | 1-7 days | 187 | 6 | 0.03 | 0.01 | 0.06 |
|  | 8-14 days | 10 | 5 | 0.50 | 0.19 | 0.81 |
|  | 15-21 days | 11 | 7 | 0.64 | 0.35 | 0.92 |
|  | 22-28 days | 4 | 4 | 1.00 | 1.00 | 1.00 |
|  | 29-35 days | 6 | 4 | 0.67 | 0.29 | 1.04 |
|  | 36-42 days | 9 | 8 | 0.89 | 0.68 | 1.09 |
|  | 43-49 days | 2 | 2 | 1.00 | 1.00 | 1.00 |
|  | 50-56 days | 6 | 5 | 0.83 | 0.54 | 1.13 |
|  | 57-63 days | 0 | 0 | NA | NA | NA |
|  | 64-70 days | 2 | 1 | 0.50 | -0.19 | 1.19 |
|  | 71-77 days | 1 | 1 | 1.00 | 1.00 | 1.00 |
|  | 78-84 days | 1 | 1 | 1.00 | 1.00 | 1.00 |
|  | 85-91 days | 2 | 2 | 1.00 | 1.00 | 1.00 |
|  | 92+ days | 8 | 6 | 0.75 | 0.45 | 1.05 |
| **Overall** |  | 1015 | 180 | 0.18 | 0.15 | 0.20 |
