# Supplementary material for: Determining Distinct Suicide Attempts From Recurrent Electronic Health Record Codes: Classification Study
Source: JMIR Form Res. 2024 Jan 8;8:e46364. doi: 10.2196/46364 (PMC10804255; doi:10.2196/46364)
Supplement: Multimedia Appendix 6 [file formative_v8i1e46364_app6.docx]

**Sensitivity Analysis (Results for Broad Sample)**

**Table S9.** Code pairs in the Broad Sample defined by the clinical settings (ED or Non-ED) of the first and second codes in each pair.

| **First code clinical setting** | **Second code clinical setting** | **Number of code pairs (% of all code pairs)** | **Median interval between codes (days)** | **Mean interval between codes (days)** | **Code pairs referring to distinct attempts** | **PPV**  **(95% CI)** |
| --- | --- | --- | --- | --- | --- | --- |
| **Non-ED** | **Non-ED** | 160 (53.33%) | 1 | 13.5  (SD: 56.1) | 9 | 0.06  (0.02, 0.09) |
| **ED** | **Non-ED** | 45 (15.00%) | 1 | 15.1  (SD: 583.6) | 6 | 0.13  (0.03, 0.23) |
| **Non-ED** | **ED** | 9 (3.00%) | 91 | 687.4  (SD: 1,022.6) | 9 | 1.00  (1.00, 1.00) |
| **ED** | **ED** | 86 (28.67%) | 80.5 | 290.7  (SD: 562.9) | 62 | 0.71  (0.63, 0.82) |
| **Overall** | | 300 | 11 | 134.3  (SD: 440.4) | 86 | 0.29  (0.24, 0.34) |

**Table S10.** Code pairs in the Broad Sample defined by whether the first and second codes referred to the same or a different suicide attempt method.

| **First and second code** | **Number of code pairs (% of all code pairs)** | **Code pairs referring to distinct attempts** | **PPV**  **(95% CI)** |
| --- | --- | --- | --- |
| **Same method** | 231 (77.00%) | 63 | 0.27  (0.22, 0.33) |
| **Different method** | 69 (23.00%) | 23 | 0.33  (0.22, 0.44) |
| **Overall** | 300 | 86 | 0.29  (0.24, 0.34) |

**Table S11.** Code pairs in the Broad Sample defined by inter-code interval.

| **Inter-code interval** | **Number of code pairs (% of all code pairs)** | **Code pairs referring to distinct attempts** | **PPV**  **(95% CI)** |
| --- | --- | --- | --- |
| **1-7 days** | 200  (66.67%) | 10 | 0.05  (0.01 – 0.09) |
| **8-14 days** | 13  (4.33%) | 6 | 0.46  (0.19 – 0.73) |
| **15-21 days** | 8  (2.67%) | 1 | 0.12  (-0.10 – 0.34) |
| **22-28 days** | 5  (1.67%) | 3 | 0.60  (0.17 – 1.03) |
| **29-35 days** | 2  (0.67%) | 1 | 0.50  (-0.19 – 1.19) |
| **36-42 days** | 4  (1.33%) | 3 | 0.75  (0.32 – 1.18) |
| **43-49 days** | 3  (1.00%) | 3 | 1.00  (1.00 – 1.00) |
| **50-56 days** | 2  (0.67%) | 2 | 1.00  (1.00 – 1.00) |
| **57-63 days** | 1  (0.33%) | 1 | 1.00  (1.00 – 1.00) |
| **64-70 days** | 0  (0.00%) | 0 | NA |
| **71-77 days** | 2  (0.67%) | 1 | 0.50  (-0.19 – 1.19) |
| **78-84 days** | 2  (0.67%) | 2 | 1.00  (1.00 – 1.00) |
| **85-91 days** | 3  (1.00%) | 3 | 1.00  (1.00 – 1.00) |
| **92+ days** | 55  (18.33%) | 50 | 0.91  (0.83 – 0.99) |
| **Total** | 300 | 86 | 0.29  (0.24 – 0.34) |

**Figure S1.** PPVs for interval floors by code pair types defined by clinical setting in the Broad Sample. The labeled data points indicate the interval floor at which the PPV was at least 0.90 (or the maximum PPV).


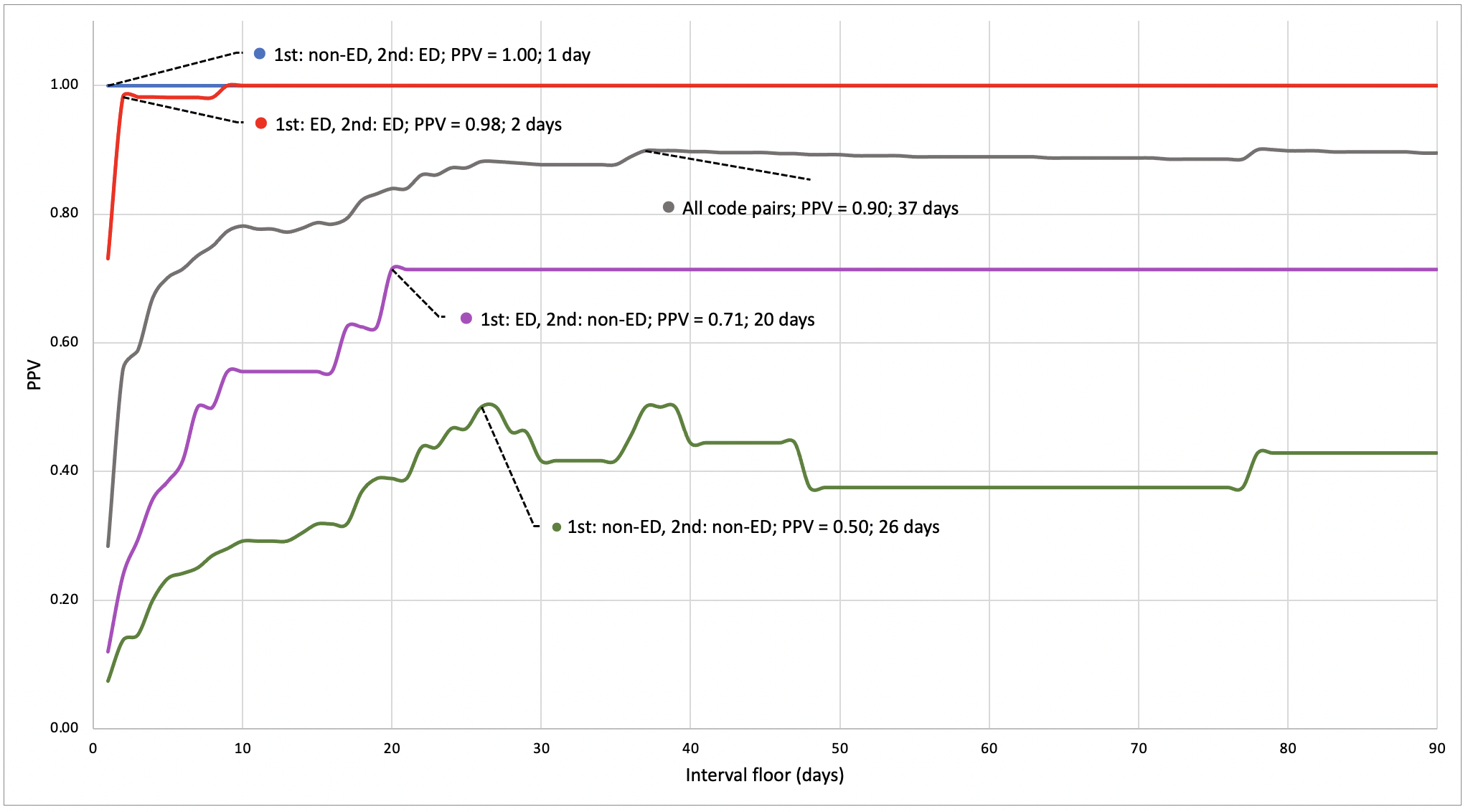


*Note.* Gray lines reflect PPVs for interval floors across all code pair types. Red lines refer to code pairs documented in ED (first code) and ED (second code) settings (ED/ED). Blue lines are Non-ED/ED code pairs; purple lines ED/Non-ED; and green lines Non-ED/Non-ED.

**Figure S2.** PPVs for interval floors by code pair types defined by suicide attempt method with intervals greater than or equal to the plotted interval floor values in the Broad Sample. The labeled data points indicate the interval floor at which the PPV was at least 0.90 (or the maximum PPV).


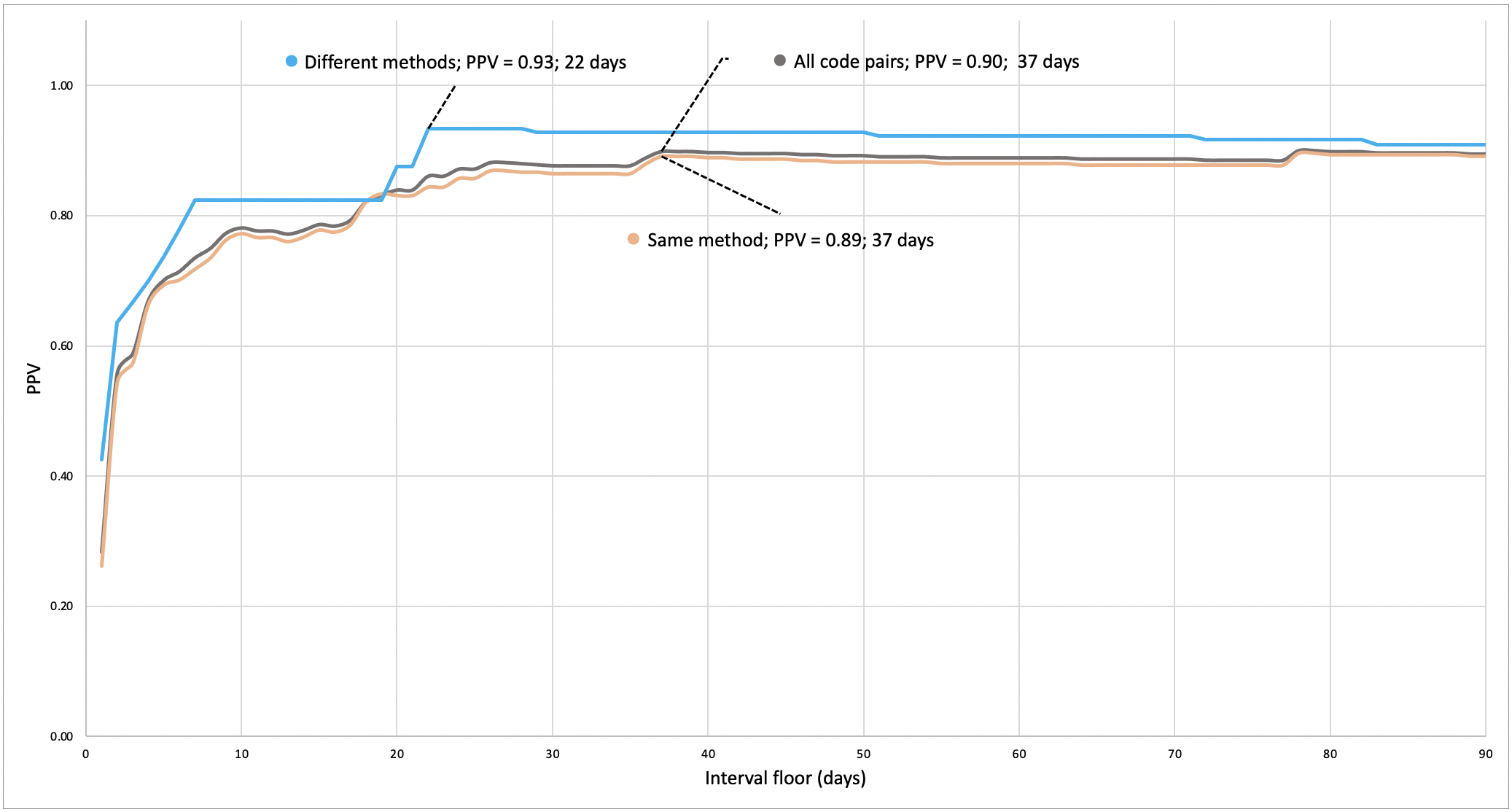


*Note.* Gray lines reflect PPVs for interval floors across all code pair types. Orange lines refer to code pairs in which the 2 codes refer to the same suicide attempt method. Blue lines refer to code pairs in codes in the pair refer to different suicide attempt methods.
